# Musculoskeletal symptoms and work ability in a context of electronic judicial process

**DOI:** 10.47626/1679-4435-2021-497

**Published:** 2021-04-30

**Authors:** Fauzi El Kadri-Filho, Thaís Moreira São-João, Neusa Maria Costa Alexandre, Roberta Cunha Matheus Rodrigues, Marília Estevam Cornélio

**Affiliations:** 1 School of Nursing, Universidade Estadual de Campinas, Campinas, SP, Brazil

**Keywords:** occupational health, musculoskeletal pain, work performance, public sector employees

## Abstract

**Introduction:**

The recent transformations undergone by Brazilian labor court, especially with the introduction of electronic process of law (*processo judicial eletrônico* [PJe]), had a significant influence on how people work.

**Objectives:**

This study aimed to evaluate the occurrence of musculoskeletal symptoms and work ability in public sector employees working in a specialized labor court body.

**Methods:**

A cross-sectional study was conducted with 449 workers, who provided demographic and occupational information and completed the Nordic Musculoskeletal Questionnaire (NMQ) and the Work Ability Index (WAI).

**Results:**

Symptoms occurred more frequently in wrists/hands (62.4%), shoulders (62.1%), and neck (60.4%) in the past 12 months, and in the neck (29.8%), shoulders (29.4%), and wrists/hands (29.2%) in the past 7 days. The mean WAI score was 38.7 (6.4), and 31.4% of participants had poor or moderate work ability. WAI scores were poorer when participants had previous problems, and the number of body segments involved in complaints was greater among those with inadequate work ability.

**Conclusions:**

Higher frequency of musculoskeletal symptoms in wrists/hands, shoulders, and neck may be related to using PJe for work and is associated with poorer work ability scores, highlighting the importance of preventive interventions for work-related musculoskeletal disorders.

## INTRODUCTION

The introduction of electronic judicial process (*processo judicial eletrônico*, PJe) has produced changes in Brazilian labor court that had a significant impact on how people work.^1^ Computerization of work can lead to increased workloads and repetitive and monotonous tasks, as well as a greater frequency of static postures and decreased movement variability. This results in increased musculoskeletal complaints in the neck and upper limbs,^2^ regardless of postural adjustments and use of ergonomically designed furniture and equipment.^3^ Increased daily time spent using the computer is related to occurrence of musculoskeletal symptoms and disorders, especially in the upper limbs and neck.^4-8^

Although studies assessing the relationship between musculoskeletal symptoms and computer use address manifestations in active workers, the reported symptoms may represent the initial stage of work-related musculoskeletal disorders (WMSDs) and lead to productivity loss in those who complain^9^ or even absenteeism.^10^ As symptoms may be related to the job, working without due clinical and/or occupational health monitoring may lead to deterioration of health, with progressive loss of work ability.^11^

Work ability can be understood as how well workers are doing at this time or will be doing in the near future and how well they are able to do their job according to the demands of their health status and physical and mental abilities.^12^ Musculoskeletal symptoms are related to reduced productivity in administrative workers and reduced work ability.^13-15^ Within this context, this study aimed to assess the occurrence of musculoskeletal symptoms and work ability in labor court employees after the introduction of PJe.

## METHODS

The study was conducted in a Regional Labor Court (Tribunal Regional do Trabalho, TRT) that has 164 trial court units in more than 100 cities in the state of São Paulo, Brazil, whose employees participated in this investigation. Employees assigned to 148 trial court units who worked exclusively in labor court secretariats, labor court outposts, and itinerant labor courts, in addition to judge’s assistants, were included. Bailiffs assigned to labor courts were not included because they carry out external activity (proceedings), as well as employees assigned to units that have regular supervised workplace exercise, whose offer was limited to two labor courthouses (16 labor courts) at the time. Considering that workplace exercise has been widely used in recent decades as a method for prevention and treatment of WMSDs, the inclusion of those workers would produce a selection bias. Employees working for less than 1 year in their units and those whose main role was being hearing secretary were excluded. Data from the participants who after having agreed to participate in the study did not fully complete the data collection instruments were not analyzed.

Sample calculation was based on the method for estimating a sample size for a proportion, considering a proportion p = 0.50, whose value represents the maximum variability of the binomial distribution, thus generating an estimate with the largest possible sample size. Considering the study eligibility criteria and the details of the information related to the distribution of positions and roles of trial court employees, the population used for sample calculation was 1564 subjects. A sampling error of 5% and a significance level of 5% were assumed. A sample size of 309 employees was then obtained, reaching at least 371 employees when taking a loss rate of 20% into account.

**Figure 1:**
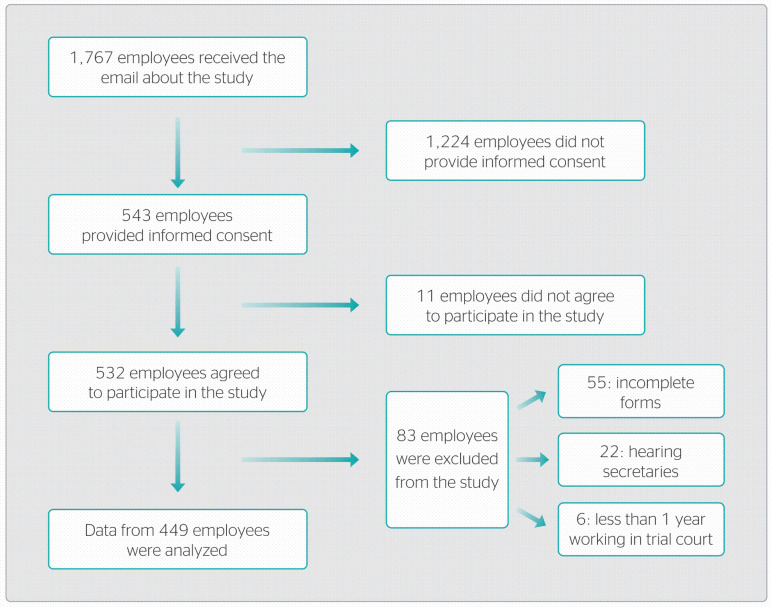
Flowchart showing selection and adherence of study participants.

In total, 1767 employees working in 148 trial court units were contacted about the study, and 543 clicked on the link sent by email and completed an informed consent form. Of those, 11 did not agree to participate. Among the 532 employees who agreed to participate in the study, 55 were excluded for incomplete data collection instruments; 22, for main role as hearing secretary; and six, for working for less than 1 year in trial court (Figure 1). Therefore, data from 449 participants working in 138 trial court units were analyzed. Response rate was 28.7%, and employees from 93.2% of units participated in the study.

Data were collected in October and November 2017 using self-administered instruments available via the Internet (SurveyMonkey®, an online survey development platform). Employees were invited to participate in the survey by contact via institutional email (with a link to access the questionnaires) and telephone call with an employee from each unit. A demographic and occupational characterization questionnaire was developed specifically for this study and consisted of questions regarding age, sex, time working at the institution, time working in trial court, average daily workload, average daily workload using PJe, and presence of diseases causing musculoskeletal symptoms in the past 12 months.

Occurrence of musculoskeletal symptoms was assessed using the Nordic Musculoskeletal Questionnaire (NMQ), which was created by Finnish researchers to standardize the evaluation of musculoskeletal symptoms in an occupational context.^16^ The Brazilian version of the NMQ was validated by Pinheiro et al.;^17^ then it was culturally adapted to the Portuguese language and had its reliability confirmed by Barros & Alexandre.^18^ The NMQ consists of questions related to presence of symptoms in the neck, shoulders, upper back, elbows, wrists and hands, lower back, hips and thighs, knees, ankles, and feet in the past 12 months and in the past 7 days. It also assesses the repercussion of symptoms on the performance of functional activities and on the need to consult a health professional. NMQ results provide a measure of how often problems occur in each body region and how many body segments involved in complaints each individual has.

Work ability was assessed using the Work Ability Index (WAI),^12^ which has shown satisfactory measurement properties regarding construct validity, criterion validity, and reliability for assessing work ability in individual approaches and population surveys in the Brazilian population.^19^ The WAI is an instrument that assesses work ability from the worker’s perception using 10 questions synthesized in seven dimensions: current work ability compared with the lifetime best; work ability in relation to the demands of the job; number of current diseases reported by the worker and diagnosed by a physician, obtained from a list of 51 diseases; estimated work impairment due to diseases; sick leave during the past year; own prognosis of work ability 2 years from now; and mental resources. Responses to item 2, regarding demands of the job, were weighted considering fundamentally mental demands, as guided by Tuomi et al.^12^ for administrative work. The scores of the seven WAI dimensions provide a measure of work ability ranging from 7 to 49 points, classified as follows: 7 to 27, poor; 28 to 36, moderate; 37 to 43, good; and 44 to 49, excellent.^19^ The first two categories can also be grouped together and classified as “inadequate work ability” (score < 37), while the last two categories can be classified as “adequate work ability” (score ≥ 37).^20,21^

Descriptive analyses of data and comparison tests were performed. The Mann-Whitney U test was used to compare WAI scores according to presence of problems in each body region and to compare the number of body segments involved in complaints according to WAI scores categorized as “inadequate” and “adequate.” Level of significance was set at p = 5%, and SAS, version 9.4, was the software used to perform the analyses.

This study was conducted in accordance with Brazilian National Health Council (Conselho Nacional de Saúde, CNS) Resolution no. 466/12 and additional regulations. The research project was authorized by TRT management and obtained approval from the union representing the employees. It was then reviewed by the Research Ethics Committee at State University of Campinas (Universidade Estadual de Campinas, UNICAMP) and approved under opinion no. 2.021.746/2017. Participants only adhered to the study and had their data used after providing consent.

## RESULTS

The mean age of the sample was 45.0 (8.4) years, and the mean time working at TRT and in trial court was 15.1 (8.6) and 14.8 (8.5) years, respectively. Most participants were female (58.8%), held the position of judicial technician (67.3%), had no specific role in the labor court (54.1%), and had no disease diagnosed in the past 12 months causing musculoskeletal symptoms (82%). The mean daily workload was 7.6 (0.9) hours, and 6.3 (1.4) hours were spent using the PJe (Table 1).

**Table 1:** Descriptive analysis of demographic and occupational variables (n = 449), state of São Paulo, 2017

Variable	n (%)	Mean (SD)	Median (IQR)	Range
Age (years)		45.0 (8.4)	46.0 (13.0)	26.0-66.0
Sex				
Female	264 (58.8)			
Male	185 (41.2)			
Position				
Executor	53 (11.2)			
Judicial technician	302 (67.3)			
Judicial analyst	94 (20.9)			
Role				
Director	62 (13.8)			
Director's assistant	47 (10.5)			
Judge's assistant	45 (10.0)			
Calculation assistant	52 (11.6)			
Other	243 (54.1)			
Time working at TRT (years)		15.1 (8.6)	15.0 (16.0)	1.0-38.0
Time working in trial court (years)		14.8 (8.5)	14.0 (16.5)	1.0-38.0
Mean workload (hours)		7.6 (0.9)	7.0 (1.0)	6.0-12.0
Mean workload using PJe (hours)		6.3 (1.4)	6.5 (1.0)	1.0-11.0
Up to 5	92 (20.5)			
More than 5 up to 6	112 (24.9)			
More than 6 up to 7	177 (39.4)			
More than 7	68 (15.1)			
Diseases in the past 12 months				
No	369 (82.0)			
Yes	80 (18.0)			

IQR = interquartile range; PJe = processo judicial eletrônico (electronic judicial process); SD = standard deviation; TRT = Tribunal Regional do Trabalho (Regional Labor Court).

Regarding NMQ results by body segment, symptoms appeared more frequently in wrists/hands (62.4%), shoulders (62.1%), and neck (60.4%) in the past 12 months, and in the neck (29.8%), shoulders (29.4%), and wrists/hands (29.2%) in the past 7 days. Shoulder symptoms were more frequently reported as responsible for limiting daily activities and for consulting a health professional (19.2% and 26.9%, respectively) (Table 2).

**Table 2:** Distribution of musculoskeletal complaints by body region (n = 449), state of São Paulo, 2017

Variable	Problems in the past 12 months n (%)	Limitations in the past 12 months n (%)	Consultations in the past 12 months n (%)	Problems in the past 7 days n (%)
Neck	271 (60.4)	82 (18.3)	107 (23.8)	134 (29.8)
Shoulders	279 (62.1)	86 (19.2)	121 (26.9)	132 (29.4)
Upper back	247 (55.0)	59 (13.1)	180 (24.1)	111 (24.7)
Elbows	130 (29.0)	37 (8.2)	57 (12.7)	60 (13.4)
Wrists/hands	280 (62.4)	81 (18.0)	101 (22.5)	131 (29.2)
Lower back	239 (53.2)	81 (18.0)	106 (23.6)	117 (26.1)
Hip/thighs	111 (24.7)	35 (7.8)	57 (12.7)	48 (10.7)
Knees	132 (29.4)	49 (10.9)	74 (16.5)	54 (12.0)
Ankles/feet	112 (24.9)	44 (9.8)	57 (12.7)	56 (12.5)

The mean WAI score of the sample was 38.7 (6.4). According to the WAI classification, 31.4% of participants had poor or moderate (inadequate) work ability, while 68.6% had good or excellent (adequate) work ability (Table 3).

**Table 3:** Frequency according to the Work Ability Index classification (n = 449), state of São Paulo, 2017

WAI	n	%
Poor	27	6.0
Moderate	114	25.4
Good	186	41.4
Excellent	122	27.2

WAI = Work Ability Index.

When comparing WAI scores with NMQ results for presence or absence of problems in nine body regions, both in the past 12 months and in the past 7 days, WAI scores were significantly higher in the absence of problems in all items (Table 4).

**Table 4:** Comparison of work ability scores according to presence or absence of problems in each body segment (n = 449), state of São Paulo, 2017

Variable	Response	n	Median (IQR)	Range	p-value*
Problems in the past 12 months in					
Neck	No	178	41.8 (7.5)	26.0-49.0 14.5-49.0	< 0.0001
	Yes	271	38.5 (9.5)		
Shoulders	No	170	41.5 (7.5)	24.5-49.0 14.5-49.0	< 0.0001
	Yes	279	38.5 (9.0)		
Upper back	No	202	42.0 (7.5)	24.5-49.0 14.5-49.0	< 0.0001
	Yes	247	38.0 (9.5)		
Elbows	No	319	40.5 (8.0)	14.5-49.0 23.0-49.0	< 0.0001
	Yes	130	36.8 (10.0)		
Wrists/hands	No	169	41.5 (8.0)	19.5-49.0 14.5-49.0	< 0.0001
	Yes	280	38.5 (9.5)		
Lower back	No	210	41.5 (7.5)	26.0-49.0 14.5-49.0	< 0.0001
	Yes	239	38.0 (10.0)		
Hip/thighs	No	338	40.5 (8.5)	19.5-49.0 14.5-49.0	< 0.0001
	Yes	111	36.5 (8.5)		
Knees	No	317	40.0 (7.5)	24.5-49.0 14.5-48.0	< 0.0001
	Yes	132	37.0 (10.7)		
Ankles/feet	No	337	40.5 (7.5)	24.5-49.0 14.5-48.0	< 0.0001
	Yes	112	35.3 (9.0)		
Problems in the past 7 days in					
Neck	No	315	41.0 (8.0)	19.5-49.0 14.5-48.5	< 0.0001
	Yes	134	36.0 (9.5)		
Shoulders	No	317	40.5 (7.5)	19.5-49.0 14.5-49.0	< 0.0001
	Yes	132	36.0 (10.0)		
Upper back	No	338	40.5 (8.5)	19.5-49.0 14.5-49.0	< 0.0001
	Yes	111	36.0 (9.5)		
Elbows	No	389	40.0 (8.5)	14.5-49.0 23.5-48.0	< 0.0001
	Yes	60	35.0 (10.8)		
Wrists/hands	No	318	40.5 (8.5)	19.5-49.0 14.5-48.0	< 0.0001
	Yes	131	37.0 (10.5)		
Lower back	No	332	40.5 (8.5)	21.0-49.0 14.5-49.0	< 0.0001
	Yes	117	37.0 (9.5)		
Hip/thighs	No	401	40.0 (8.5)	19.5-49.0 14.5-48.0	< 0.0001
	Yes	48	34.0 (9.0)		
Knees	No	395	40.0 (8.5)	19.5-49.0 14.5-47.0	< 0.0001
	Yes	54	34.8 (9.5)		
Ankles/feet	No	393	40.0 (8.5)	19.5-49.0 14.5-44.5	< 0.0001
	Yes	56	34.0 (9.0)		

IQR = interquartile range.

* Mann-Whitney U test.

WAI scores were categorized as “inadequate” or “adequate.” When these two categories are compared, the number of body segments involved in complaints showed a statistically significant difference, with better results being observed in the “adequate” category for all questions (Table 5).

**Table 5:** Comparison between occurrence of musculoskeletal symptoms (number of body segments involved in complaints) according to Work Ability Index category (n = 449), state of São Paulo, 2017

Variable	WAI	n	Median (IQR)	Range	p-value*
Problems in the past 12 months	< 37	146	5.0 (3.0)	0.0-9.0 0.0-9.0	< 0.0001
	≥ 37	303	3.0 (3.0)		
Limitations in the past 12 months	< 37	146	2.0 (4.0)	0.0-9.0 0.0-8.0	< 0.0001
	≥ 37	303	0.0 (1.0)		
Consultations in the past 12 months	< 37	146	3.0 (4.0)	0.0-9.0 0.0-9.0	< 0.0001
	≥ 37	303	0.0 (2.0)		
Problems in the past 7 days	< 37	146	3.0 (4.0)	0.0-9.0 0.0-9.0	< 0.0001
	≥ 37	303	1.0 (2.0)		

IQR = interquartile range; WAI = Work Ability Index.

* Mann-Whitney U test.

## DISCUSSION

Symptom occurrence in the past 12 months had results similar to those of the study conducted by Griffiths et al.,^4^ in which complaints were collected using the WAI; thus, in general, the same body regions were among the most affected in activities involving intensive typing (neck, shoulders, and wrists/hands). However, Griffiths et al. found a greater prevalence of symptoms in the neck and shoulders (80% and 79%, respectively), followed by complaints in the lower back (66%) and wrists/hands (58%). These results are probably related to Griffiths et al. having evaluated workers with different roles, whereas the present study evaluated workers with similar roles. Additionally, there was less variation in the time spent using the computer in the study sample, given that the mean percentage of PJe use during daily working hours reached 83.4% at the time of data collection.

Other studies evaluating musculoskeletal symptoms among workers used different instruments and NMQ adaptations, which makes direct comparison of results difficult. Nonetheless, in most of them there were more complaints in the neck, shoulders, and, especially, wrists/hands with the intensification of computer use at work.^2,7,8^ The higher number of complaints in the neck and upper limbs may be related to increased workloads and monotonous tasks resulting from the computerization of work processes, which were, in turn, related to a greater frequency of static postures and decreased movement variability.^4,6^ Symptoms in the neck, shoulders, and wrists/hands were also among the complaints that were most frequently reported as responsible for limiting daily activities and for consulting a health professional, which is consistent with the relationship with the job performed by study participants and should generally represent more severe symptoms in those body segments.

The mean WAI score of the sample, even with the inclusion of participants who reported diseases causing musculoskeletal symptoms, was 38.7 (6.4), therefore within the spectrum of good work ability.^22^ This result was very close to those obtained by Gharibi et al.,^23^ who reported a mean WAI score of 38.0 (6.3) in workers of different sectors, and by Guidi et al.,^24^ who found a mean score of 38.0 (6.27) in bank employees. However, the result is well below the mean score of 42.7 (4.2) obtained by Monteiro & Fernandes^25^ in workers of an information technology and telecommunications company.

While approximately 1/3 of the participants in the present study had inadequate work ability, more than 2/3 had adequate work ability. Conversely, a study of Pernambuco Court of Justice employees conducted by Santos et al.^26^ found a greater proportion of participants in the poor or moderate work ability group (37.5%), while 62.5% of the employees showed good or excellent ability, with a mean WAI score of 37.6 (4.5), similar to that of the present study.

A study of Pernambuco TRT employees^27^ showed that only 22% of participants had poor or moderate work ability and 78% had good or excellent ability, with a mean score of 40.0 (5.0). That study sample had demographic and occupational characteristics similar to those of the present study in terms of mean age and time working at the TRT; however, there were differences regarding the inclusion of administrative employees and, especially, regarding the period of data collection, which was conducted in 2011, ie, prior to the implementation of PJe in labor court.

The relationship between occurrence of musculoskeletal symptoms and work ability is clear when WAI scores are compared according to the presence of problems in each body segment and when the number of body segments involved in complaints among the participants is considered for each NMQ question. In the first case, significant differences were obtained in the median WAI scores for all body segments, according to the presence or absence of problems in the past 12 months and in the past 7 days. In the second, the median number of body segments involved in complaints among participants with adequate work ability was significantly higher than among those with inadequate ability. This relationship can be understood to the extent that musculoskeletal function is among the factors that have most impact on functional ability, which is seen as the basis for work ability as it influences how weary the worker is and is related with performance of job demands.^20^ Indeed, among the studies that used the WAI to establish a relationship with musculoskeletal complaints, a lower number of symptoms was related to better work ability scores.^21,22,28^

Regarding the impact of musculoskeletal symptoms on work ability, it is worth noting that work ability is a complex structure affected by a set of different interactions between biological aging, health, skills, organizational context, social context, and demands of the job. Work ability has been influenced by self-reported musculoskeletal symptoms and perception of health-related variables. From this perspective, specific health promotion strategies should be designed and implemented to contribute to preserving work ability over the years.^29^

Regarding healthy worker bias associated with workers that were on sick leave, on vacation, or retired having not been included in the sample, it should be considered that active workers also have diseases or health conditions that are consistent with work routine, such as presence of noncommunicable diseases and others. From this perspective, all participants included in the sample had the same chances of having their perceived symptoms or work ability modified by the presence of conditions that moderate their responses. A study^30^ evaluating 1686 Chinese workers of sectors such as administration and education divided the sample between those with and those without some WMSD and found a significant difference between groups, with decreased work ability among those who had some disorder.

Although the total number of participants was high and the minimum sample size was exceeded, the response rate obtained could better represent the study population if it were higher. Employees from units that had supervised workplace exercise were not included in this study because of the well-documented relationship between that practice and prevention of musculoskeletal symptoms and the possibility of producing, therefore, a selection bias, given that more than 90% of the units had no such activity. However, this is a factor that may have contributed to poorer NMQ and WAI results in our sample.

Collecting data via the Internet, at the same time that it favors the participation of workers who are geographically dispersed, may disfavor their engagement in the study. Similarly, the fact that participants reported both symptom occurrence and work ability (both self-reported measures) may have produced a single source bias.

Because this is a cross-sectional study, even though important relationships between study variables were observed, defining cause-effect relationships was not possible. One possibility is that, considering the nature of the study variables, poorer work ability scores may be related to increased musculoskeletal complaints, resulting in a reverse causality bias. To that end, new longitudinal studies should be conducted with this population for a more adequate analysis of those relationships.

In conclusion, the present study aimed to assess the occurrence of musculoskeletal symptoms and their relationship with work ability in labor court employees in the context of PJe. The findings suggest that the occurrence of musculoskeletal symptoms, having been higher in the wrists/hands, shoulders, and neck, may be related to using PJe for work and is associated with poorer work ability scores. This relationship highlights the importance of preventive WMSD-related interventions motivated not only by absenteeism rates, given that the occurrence of musculoskeletal symptoms in active workers, in addition to affecting their well-being, is also capable of affecting performance and productivity.
